# Protocol and baseline data for a prospective open-label explorative randomized single-center comparative study to determine the effects of various intravenous iron preparations on markers of oxidative stress and kidney injury in chronic kidney disease (IRON-CKD)

**DOI:** 10.1186/s13063-019-3291-x

**Published:** 2019-04-04

**Authors:** Ahmed Ziedan, Sunil Bhandari

**Affiliations:** Hull University Teaching Hospitals NHS Trust and Hull York Medical School, Hull Royal Infirmary, Anlaby Road, Hull, HU3 2JZ UK

**Keywords:** Acute kidney injury, Chronic kidney disease, Cosmofer, Intravenous iron, Iron deficiency, Monofer, Oxidative stress, Protocol, Randomized trial, Venofer

## Abstract

**Background:**

Intravenous (IV) iron is frequently used to treat iron deficiency/anemia in patients who are unable to tolerate oral iron or the oral iron is not sufficient toreplete iron requirements. However, safety concerns regarding the potential increase in oxidative stress and other adverse effects persist and it remains unclear whether all iron preparations are equivalent. Indeed, the comparative risk of adverse events with IV iron preparations has not been extensively assessed.

We hypothesize that IV iron leads to changes in oxidative stress, endothelial function, and potential renal damage depending on the iron formulation (related to the generation of “free” or catalytic labile iron) and this may result in more tubular and glomerular injury manifested as increased proteinuria and raised neutrophil gelatinase–associated lipocalin (NGAL) levels in patients with chronic kidney disease (CKD).

**Methods:**

IRON-CKD is a prospective, open-label, explorative, randomized, single-center study designed to compare the safety and efficacy of three parenteral iron preparations: low-molecular-weight iron dextran–Cosmofer, iron sucrose–Venofer, and iron isomaltoside–Monofer. The study includes 40 adults who have established CKD stages 3–5 and serum ferritin (SF) of less than 200 μg/L or transferrin saturation (TS) of less than 20% (or both); they were randomly assigned in a 1:1:1:1 ratio to 200 mg iron dextran, 200 mg iron sucrose, 200 mg iron isomaltoside, or 1000 mg iron isomaltoside. After randomization, participants undergo baseline assessments and then an iron infusion. Each participant is followed up at 2 h, day 1, week 1, and months 1 and 3. At each follow-up visit, patients undergo clinical review, measurement of pulse wave velocity (PWV), blood tests for renal function, and collection of serum/plasma samples for oxidative stress and inflammatory markers.

The primary outcomes are measures of oxidative stress, inflammatory markers, and markers of acute renal injury in comparison with baseline measures of each iron preparation and between each of the iron preparations. Secondary objectives include effects on hematinic profiles and hemoglobin concentrations, changes in arterial stiffness, incidence of significant side effects, and change in patients’ quality of life.

**Results:**

Between October 2015 and April 2018, 521 individuals were identified as potential participants; 216 were contacted, 56 expressed an interest, 49 attended a screening visit, and 40 were confirmed to meet the eligibility criteria and were randomly assigned. The mean age was 58.3 (standard error of the mean 4.4) years, and 23 (58%) were male. All patients were white and English-speaking. The mean SF was 66.6 μg/L, TS was 21.2%, and hemoglobin was 121.6 g/L at randomization for the whole group. The mean estimated glomerular filtration rate was 27.8 mL/min, the urinary protein/creatinine ratio was 104.3 mg/mmol, and CRP was 6.65 mg/L.

**Discussion:**

IRON-CKD will provide important information on the short-term effects of three preparations of IV iron in CKD patients with biochemical functional or absolute iron deficiency on measures of oxidative stress, inflammation, endothelial function, and renal injury.

**Trial registration:**

European Clinical Trials Database (EudraCT) number 2010-020452-64.

## Background

Chronic kidney disease (CKD) is a worldwide public health problem that affects about 4–6% of the UK adult population and is associated with a high prevalence of cardiovascular disease and high economic cost [1]. Patients have either absolute (depletion of both circulating and iron stores) or functional (depletion of circulating/available iron) iron deficiency. Currently, intravenous (IV) iron is used regularly in patients with CKD to correct anemia and optimize the use of erythropoietin-stimulating agents [2, 3].

Debate remains regarding the effects of IV iron on oxidative stress, renal function, and proteinuria in patients with CKD. Clinical data on the effects on renal function are encouraging [4]. However, assessment of transient damage and markers of potential damage are required to confirm these clinical findings.

Critical to the importance of iron in biological processes is its ability to cycle reversibly between its ferrous and ferric oxidation states. This precise property, which is essential for its functions, also makes it dangerous because “free” (labile) iron can catalyze the formation of free radicals leading to cell damage. Labile iron (catalytic iron) consists of chemical forms that can participate in redox cycling. This property makes iron potentially hazardous by enabling it to participate in the generation of powerful oxidant species (such as hydroxyl radical) (metal-catalyzed Haber–Weiss reaction) or reactive iron–oxygen complexes (such as ferryl or perferryl ion) or both [5]. Iron also has a major role in the initiation and propagation of lipid peroxidation by either catalyzing the conversion of primary oxygen radicals to hydroxyl radicals or forming a perferryl ion. This pool of labile iron is increased in many disease states. Certain iron chelators may provide a protective effect, thus establishing a cause–effect relationship, at least in animal models [6]. Several animal models have demonstrated the potential detrimental effects of “free” iron on glomerular function [7]. However, there are limited human data. Shah *et al*. compared the effects of catalytic iron in subjects with either no renal disease or diabetes with patients with diabetes and managed to demonstrate that patients with overt diabetes have an increase in urinary catalytic iron [6]. In their preliminary studies, the authors showed that treatment with the chelator deferiprone leads to a reduction in proteinuria in patients with diabetic nephropathy and glomerular diseases which is unresponsive to other treatments [6, 7]. Lin *et al*. have also shown that chelation therapy with ethylenediaminetetraacetic acid (EDTA) in patients with CKD results in a reduced rate of decline in the glomerular filtration rate [8]. The authors attributed the beneficial effect to the chelation of lead, which also participates in the Fenton reaction. However, given the affinity constants for iron and lead, the large experimental evidence for the role of iron in kidney disease and the demonstrated efficacy of EDTA in enhancing excretion of urinary iron suggest that the beneficial effects are more likely to be explained by the chelation of iron rather than lead [8].

When IV iron is administered, it passes to the reticulo-endothelial system (RES). The iron complex with dextran, isomaltoside, or sucrose splits. Iron is therefore combined to ferritin or transferrin, which is used in hemoglobin (Hb) production and storage. Iron dextran consists of ferric oxyhydroxide and polymerized dextran; the former passes to the RES and is eliminated from the plasma at 10–20 mg/h as it is released from the dextran complex to bind to transferrin and pass to the erythroid bone marrow [9]. Iron sucrose consists of soluble iron, hydroxide, and sucrose, which pass to the RES and then to the bone marrow. However, a small percentage of iron may be released as free iron. This may be toxic to cells, in particular glomerular and mesangial cells via oxidative stress (lipid peroxidation) and cell cytotoxicity, leading to endothelial dysfunction, which leads to proteinuria, accelerated atherosclerosis, and potentially increase in serum creatinine. This oxidative stress also leads to a reduction in ATP production via mitochondrial damage. Previously, in animal studies, Zager *et al*. have demonstrated that sucrose-based iron preparations may cause direct cytopathic changes to renal cells and this therefore potentially causes renal deterioration [10–12].

Iron isomaltoside consists of a linear and unbranched oligosaccharide carbohydrate moiety where the iron is tightly bound in a matrix structure. This enables a controlled and slow release of iron to iron-binding proteins and passage to the RES, thus avoiding potential toxicity from the release of labile iron. The strongly bound iron within the iron isomaltoside formulation allows flexible dosing over a short time period. Compared with compounds in which iron is more loosely bound in the complex, the iron isomaltoside complex potentially leads to the generation of less oxidative stress and less immunological toxicity [13].

Neutrophil gelatinase–associated lipocalin (NGAL) is a 25-kDa glycoprotein that is normally found in neutrophils, hepatocytes, and proximal tubular cells [14, 15]. Its expression is increased in conditions such as inflammation and infection. High levels of secretion into the blood and urine upon injury to the kidney are seen, for example, during renal ischemia, which occurs after cardiopulmonary bypass and in critically ill patients [16]. It has been shown that urinary NGAL and plasma NGAL are diagnostic of acute kidney injury (AKI) in critical illness with a sensitivity and specificity of more than 85% [14–18]. Therefore, NGAL may be a useful early marker of AKI. NGAL levels are increased within hours of injury. This therefore could help elucidate whether IV iron therapy potentially causes acute renal injury, which goes undetected and gives further insight into the etiology of renal injury. NGAL therefore may be a good indication of renal injury but not necessarily of renal function. Of course, NGAL is increased in the blood and urine in other conditions which may serve as confounders, such as inflammation and infection and various types of cancer. However, specific levels have been determined for these. Furthermore, the combined use of other oxidative stress and inflammatory markers will help advance the field of biomarkers of drug kidney toxicity.

### Research question

The IRON-CKD trial has been designed to assess the effects of three preparations of IV iron (Cosmofer, Venofer, and Monofer) in a cohort of CKD patients with biochemical functional or absolute iron deficiency on changes in oxidative stress, inflammation, and potentially acute effects on renal function. In addition, the differential effects of low-dose and high-dose iron isomaltoside (Monofer) on the various parameters will be assessed.

### Primary, secondary, and other objectives

As this is an “explorative” study, the primary objective is to provide valuable information on the following outcomes of interest:

1. oxidative stress and inflammatory markers
2. measures of acute renal injury, including serum creatinine, estimated glomerular filtration rate (eGFR), urinary proteinuria, and NGAL
3. measures of iron status (hematinic profiles) and response to treatment
4. Hb response to IV iron
5. effects of IV iron on endothelial function assessed by pulse wave velocity (PWV)
6. change in patients’ quality of life
7. documented acute clinical side effects as a result of iron infusion.

### Primary outcome measures

1. measures of oxidative stress
2. inflammatory markers
3. markers of acute renal injury in comparison with baseline measures and between iron preparations.

### Secondary outcome measures

1. effects on hematinic profiles
2. effects on Hb concentrations
3. changes in arterial stiffness
4. incidence of significant side effects
5. change in patients’ quality of life.

### Study design

This is an investigator-led, open-label, single-center, prospective, randomized explorative study involving patients attending nephrology outpatient clinics for a screening visit (visit 1) and five study follow-up visits, including the baseline visit.

### Ethics

This study was carried out in accordance with good clinical practice guidelines and the Declaration of Helsinki and received ethical approval from the Northern Regional Ethics Service (NRES) Committee Yorkshire and the Humber - Leeds East, UK (approval reference number 10/H1306/40). If participants should suffer harm due to the study, the trial is covered by the National Health Service (NHS).

#### Indemnity insurance

The majority of patients attending the service are white British as 92% of the geography of the region is white. Therefore, no non-white participants were recruited in view of the potential confounder with inflammatory measures and increased cost associated with translated information leaflets.

Iron replacement is initiated on the basis of a set protocol which is in accordance with Renal Association and NICE (National Institute for Health and Care Excellence) guidelines [19]. Potential participants attending the nephrology clinic in Hull Royal Infirmary were invited to take part in the study. Written information was provided and signed consent was obtained from each patient. Serum ferritin (SF), iron, and transferrin saturation have been monitored at baseline and throughout the study in accordance with protocol.

**Fig. 1:**
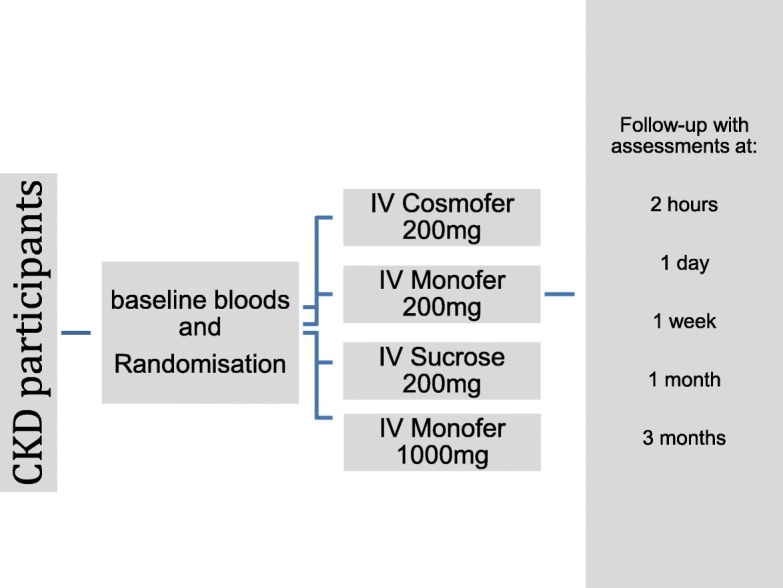
Flow chart of patient’s journey in the trial. Follow-up visits are 2 h, 1 day, 1 week, and 1 month and 3 months after iron infusion.

Conventional additional therapies were adjusted as deemed necessary for best clinical practice. Oral iron was stopped for the duration of the study. Participants were randomly allocated to receive one of four iron therapies and will be followed up at 2 h, day 1, week 1, and months 1 and 3 (Fig. 1).

### Eligibility

We recruited 40 adults participants with CKD (stages 3–5) and functional or absolute iron deficiency (SF level < 200 μg/L or transferrin saturation (TS) < 20% or both) who were due to receive therapeutic IV iron on the basis of the inclusion and exclusion criteria and who had not had an iron infusion in the last 6 weeks (Table 1). The exclusion criteria were designed to identify participants for whom the safety of iron infusion may have been a concern or those with potential confounding factors to NGAL or iron marker measurements such as cancer, infection, or hemoglobinopathy.

**Table 1:** Inclusion and exclusion criteria

Inclusion criteria	Exclusion criteria
≥18 years of age	Age < 18 years
Chronic kidney disease (CKD) stages 3–5 (glomerular filtration rate < 60 mL/min per 1.73 m^2^)	No renal failure or CKD stages 1–2
Written and signed informed patient consent and ability to co-operate with study protocol	Patients unable or do not wish to give consent or inability to co-operate with study protocol
No previous iron administration in last 6 weeks	Parenteral iron therapy within previous 6 weeks
Serum ferritin level less than 200 μg/L	Ferritin greater than 200 μg/L
Transferrin saturation < 20%	Transferrin saturation > 40%
Non-smoker or ex-smoker	Current smokers
	Hemochromatosis
	Patients with potential confounding factors to neutrophil gelatinase–associated lipocalin (NGAL) measurement (cancer, infection)
	Pregnancy
	Patients being investigated for potential blood loss
	Patients with a hematological malignancy/ hemolysis or known hemaglobinopathy, including myeloma
	Known allergy to any iron therapy

### Study enrolment and randomization

#### Identifications and invitation

Participants were identified either when presenting for routine visits or through searching the hospital electronic database for potential patients by using the inclusion/exclusion criteria. After confirming eligibility, the investigator approached eligible participants to ascertain interest. A verbal explanation of the trial and an invitation letter with a copy of the patient information sheet were mailed to interested individuals. This included information about the rationale, design, and personal implications of the study. After information was provided, participants had at least 24 h to consider whether to participate and were given the opportunity to discuss the trial with their family and health-care professionals before they were invited to attend the screening visit.

### Consent and screening

At the screening visit, eligibility was assessed and written informed consent was obtained by a medically qualified investigator who explained the questionnaire, the procedures required, risks/benefits, and confidentiality to the potential participant prior to obtaining informed consent from willing participants.

Relevant details of participants’ medical history (including primary renal diagnosis, iron deficiency status, and concurrent medications) were recorded. Oral iron was discontinued prior to randomization, and participants were instructed to notify the study site about any new medications they took after the start of the study.

After a physical examination, blood and urine samples from willing participants were sent to the hospital’s pathology laboratory for confirmation of eligibility. If the results were considered inaccurate (e.g., hemolyzed sample) by the investigator, the samples could be repeated once; but if the results did not confirm eligibility, the participant was withdrawn from the study.

### Randomization procedure

After informed consent was obtained, eligible participants were assigned a unique participant identification number. Once this number was assigned to participants, it was not reused. Participants were randomly allocated in a 1:1:1:1 ratio to receive one of the three iron preparations (and two dosing concentrations for Monofer) at visit 2. The randomization was performed by a computer program in blocks, and the block size was not revealed. Labels were consecutively numbered 1–40. Then they were sealed in non-transparent double-sealed envelopes. Access to these envelopes was not available to investigators. Details of the iron therapy were held by pharmacy that matched the choice of interventional iron therapy with the relevant randomization number. Both numbers and iron therapy treatment administered at visit 2 were recorded in the participants’ medical case records.

### Baseline measurements

After randomization, willing and eligible participants were invited to attend a baseline visit, at which baseline investigations were performed (Table 2). These included PWV, eGFR, biochemical profile (BCP), full blood count (FBC), SF, TS%, and C-reactive protein (CRP). Two further samples of plasma and serum were obtained from participants and stored at −80 °C for future analysis of NGAL, oxidative stress, and inflammatory markers at the University of Hull. Samples were kept on ice in a sealed container when transported from the hospital outpatient clinic to the hospital’s pathology laboratory or from the hospital to the university.

**Table 2:** Summary of schedule detailing all of the assessments required at each visit

Procedures	Visit 1	Visit 2a	Visit 2b	Visit 3	Visit 4	Visit 5	Visit 6
	Screen	baseline	Post iron 2 h	1 day	1 week	1 month	3 month
Informed consent	x						
Demographics	X						
History	X						
Examination	X				X	X	X
Weight	X	X		X	X	X	X
BCP	X	X	X	X	X	X	X
Albumin	X	X	X	X	X	X	X
eGFR	X	X	X	X	X	X	X
FBC	X	X	X	X	X	X	X
Ferritin	X	X	X	X	X	X	X
TS%	X	X	X	X	X	X	X
CRP	X	X	X	X	X	X	X
Cystatin C		X	X	X	X	X	X
uACR/uPCR		X	X	X	X	X	X
NGAL		X	X	X	X	X	X
Oxidative stress markers		X	X	X	X	X	X
Pulse wave velocity		X	X	X	X	X	X
SF-36		X			X	X	X
Medication check		X			X	X	X
Adverse events			X	X	X	X	X

“X” indicates action to be taken or an investigation to be performed

*Abbreviations*: *BCP* biochemical profile (consisting of urea and electrolytes, creatinine, liver function tests, albumin, total protein, calcium, phosphate, and bicarbonate), *CRP* C-reactive protein, *eGFR* estimated glomerular filtration rate, *FBC* full blood count (consisting of hemoglobin, white cell count, platelets, mean cell volume, and mean cell hemoglobin concentration), *NGAL* neutrophil gelatinase–associated lipocalin, *SF-36* 36-Item Short Form Health Survey, *TS%* transferrin saturation percentage, *uACR/uPCR* urinary albumin:creatinine ratio/urinary protein:creatinine ratio

At baseline, participants were asked to complete a preference-based measure of health (SF-36) questionnaire, a standardized survey used to assess patient health across eight dimensions [20]. After all baseline measurements were performed, participants were accompanied to the hospital’s medical day unit, where IV iron was administered in accordance with hospital policy.

### Iron administration

Participants were randomly assigned to receive one of the four options. Infusions consisting of 200 mg of the iron preparation Venofer, Cosmofer, or Monofer or an infusion of 1000 mg of Monofer were supplied by hospital pharmacy in accordance with departmental protocol. All preparations were administered over a 1-h time period apart from the low-dose Monofer, which was given over 15–20 min in accordance with normal practice at Hull and East Yorkshire Hospitals NHS Trust (renamed Hull University Teaching Hopsitals NHS Trust). Patients were closely monitored throughout the infusion and 30 min later for hemodynamic changes or side effects. Two hours after the infusion, participants were reviewed again and all investigations performed at baseline (except the health questionnaire) were repeated. Participants were monitored closely for adverse effects throughout the study and any events were recorded in the electronic case report and in the medical notes.

### Assessments

At all visits, the investigator sought the information on adverse events (serious and non-serious) considered by participants to be related to iron therapy. In addition to documenting changes in health and concurrent medications, two participants who were considered to require further iron infusions by the caring nephrologist whilst still on the study were withdrawn. Weight, blood pressure, temperature, and PWV were measured at all visits, and the SF-36 questionnaire was completed at 1-month and 3-month visits (Table 2).

Blood levels to measure Hb, iron markers, CRP, and kidney function were performed, as in normal practice. Quantification of proteinuria was carried out by measurement of urinary protein:creatinine ratio (uPCR) or, if diabetic, urinary albumin:creatinine ratio (uACR) levels in a spot urine sample by using standard laboratory techniques.

Additional samples from EDTA and serum-separating tubes were centrifuged and the plasma and serum aliquoted into cryovials, which were initially stored locally at −80 °C prior to transfer to the university laboratory in Hull, UK, where they will be stored at −80 °C until analysis for inflammatory and oxidative stress biomarkers.

After 3 months of study follow-up, participants will continue to be reviewed in accordance with normal practice three times a month. All information was collected and recorded on a secure encrypted and password-protected computer database in the research unit at the NHS Trust.

### Enzyme-linked immunosorbent assay for quantitative detection of NGAL

NGAL will be measured by enzyme-linked immunosorbent assays (Life Technologies | Thermo Fisher Scientific, Carlsbad, CA, USA) for quantitative detection of human NGAL and expressed as nanograms per milligram per creatinine. In brief, the plates are precoated with primary NGAL antibody and therefore ready to use. The calibrators and dilated sample are added to the wells and incubated for 30 min. Tetramethylbenzidine or a substitute is added to each well along with a stop solution after 15 min and then quantum results are obtained by measuring the absorbance at 450 nm.

### Oxidative stress biomarkers

Plasma malondialdehyde (MDA) levels will be measured by derivatization with thiobarbituric acid by using an isocratic high-performance liquid chromatography technique.

### F2-isoprostanes

A competitive immunoassay with colorimetric quantification (Direct 8-iso-Prostaglandin F2α Enzyme Immunoassay Kit, Assay Designs, Enzo Life Sciences, Farmingdale, NY, USA) will be used to measure 8-iso-prostaglandin F2α (8-iso-PGF2α), also termed F2-isoprostane, as a marker of lipid peroxidation.

### Non-transferrin-bound iron and labile plasma iron

Levels of non-transferrin-bound iron (NTBI) and labile plasma iron (LPI) will be measured by using the FeROS™ assay (Aferrix Ltd., Tel-Aviv, Israel)..This assay employs a selective iron chelator that blocks redox cycling of iron to specifically identify iron-mediated reactive oxygen species generation. Comparison of the fluorescence generated in the reaction in the presence and absence of the iron chelator translates into an accurate estimate of the quantity of LPI in the tested sample.

### Monitoring

Prior to recruitment, study staff received training in the study procedures. The study has been monitored in accordance with Hull and East Yorkshire Research and Development Department standard operating procedures to ensure compliance with UK Clinical Trial Regulations. Deviations from the protocol or good clinical practice were reported by the investigator to the Department (as sponsor) on monitoring report forms every two months. Investigators will take into account all protocol deviations and any serious breaches in the final study analysis and publication.

### Statistical considerations

#### Sample size calculation

As this was an explorative pilot study looking for proof of concept, a size calculation was not required. There are no previous studies examining the differential impact of different intravenous iron preparations on markers of AKI. Other studies examining AKI using NGAL, have demonstrated that a plasma NGAL (at a cutoff value of 50 μg/L) were powerful independent predictors of AKI, with an area under the receiver operating characteristic curve of 0.91 [16]. Therefore, one would anticipate a large change from baseline of NGAL values as a result of intravenous iron if the hypothesis is correct.

### Statistical analysis

All analyses will involve comparing outcomes and changes in the different parameters during the scheduled study visits among all participants randomly assigned to receive one of the four iron therapies. Comparisons of continuous outcomes (including the primary outcome) between the allocated treatment arms will be performed by using analysis of covariance (ANCOVA) adjusted for each patient’s value at baseline. Multiple imputation techniques with such a small study will be of limited value. Therefore, we will analyze all available data without data replacement as these are missing at random rather than related to the trial intervention. The safety population will include any randomly assigned participants who received any amount of study drug. Descriptive statistics and graphic approach will be employed for the exploratory analysis of variations in oxidative stress, inflammatory markers, and markers of acute kidney injury. In addition, we will explore the impact of diabetes and gender, but because of the size of this explorative study, we will examine the impact of diabetes (*n* = 13) in the whole cohort versus non-diabetes (*n* = 27) but this analysis will be simply hypothesis-generating rather than conclusive. Being on lipid-lowering medication such as statins may affect lipid peroxidation and activities of antioxidant enzymes. Broncel *et al*. [21] have shown that in patients with dyslipidemia without clinical symptoms of atherosclerosis, atorvastatin, simvastatin, and pravastatin decreased similar thiobarbituric acid-reactive substance (TBARS) concentrations in the isolated erythrocyte membranes. The authors observed a significant increase of the antioxidant enzyme activities during atorvastatin and simvastatin treatment. Therefore, this will be examined to see whether there is an impact in those patients but again numbers will be small.

## Results

**Fig. 2:**
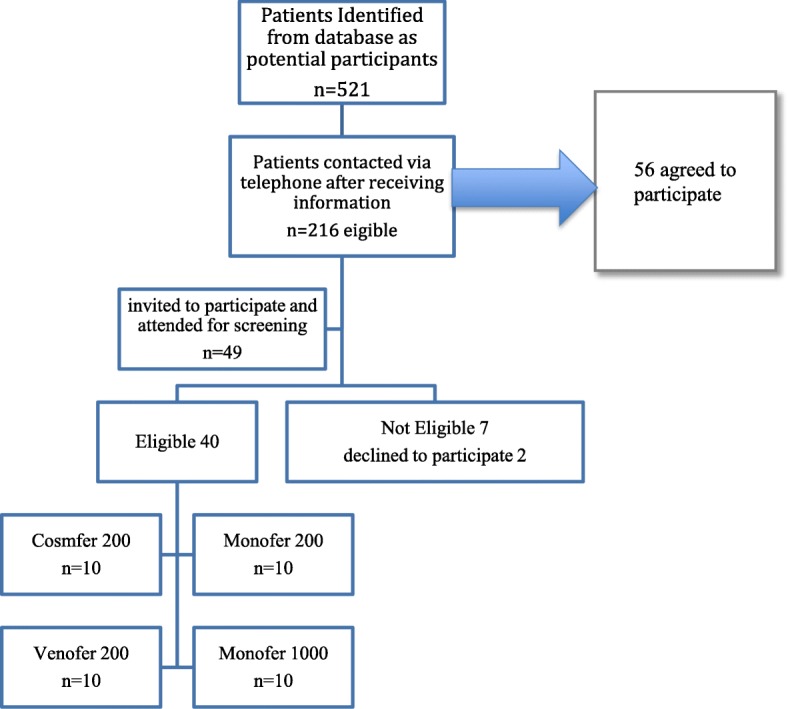
Consort figure of flow of patients through IRON-CKD trial

Between October 2015 and April 2018, 521 individuals were identified as potential patients by using lists of patients awaiting IV iron therapy and using an electronic database of patients with CKD and long-term iron deficiency; 237 (45.5%) were found to live locally. Of these, 216 (91%) were contacted by telephone after receiving an information sheet and 56 (26%) expressed interest in taking part in the study. Most of these patients lived locally with a maximum of 30-min driving time to the hospital, which has contributed to their compliance throughout the study. Another major factor that contributed to patient compliance was their mobility and access to transport (i.e., regardless their age or gender). We found that the more independently mobile the patients were and the easier their access to private or public transport was, the more likely they were to consent to take part in a study with six visits over a 3-month period. Forty-nine patients attended a screening visit, which usually coincided with their usual follow-up nephrology appointment. Seven of them did not meet the eligibility criteria following screening and this was due mainly to iron markers being normal or above the required range. Two patients felt that the study contained too many visits and therefore decided not to continue taking part. Eventually, 40 were confirmed to meet the eligibility criteria (Fig. 2). All eligible patients were randomly assigned to intervention with one of the four iron preparations and then followed up at 2 h, 1 day, 1 week, 1 month, and 3 months after the infusion.

### Baseline characteristics of randomly assigned participants

In total, 40 patients have been randomly assigned. The mean age was 58.3 years (standard error of the mean (SEM) 4.4.) and 23 (58%) were male (Table 3). The mean SF was 66.6 μg/L (SEM 15), TS 21.2% (SEM 3.2), and Hb 121.6 g/L (SEM 5.46) at randomization for the whole group. The mean eGFR was 27.8 mL/min per 1.73 m^2^ (SEM 4), uACR 110.4 mg/mmol (SEM 65.3), and uPCR 104.3 mg/mmol (SEM 67). CRP was 6.7 mg/L (SEM 2.3). The etiology of the renal failure varied between the patients and is detailed in Table 4.

**Table 3:** Baseline demographic data

	Venofer 200	Cosmofer 200	Monofer 200	Monfer 1000	Total group
Age, years	49.5 (4.3)	66.2 (2.3)	59.6 (5.0)	58.8 (5.9)	58.3 (4.4)
Male/female, numbers	6/4	6/4	4/6	7/3	23/17
Serum ferritin, μg/L	58.5 (11.6)	80.8 (20.6)	60.5 (9.5)	66.6 (18.4)	66.6 (15.0)
Transferrin saturation percentage	21.1 (2.9)	25.4 (4.9)	21.2 (3.1)	18.1 (1.7)	21.2 (3.2)
Hemoglobin	121.4 (4.8)	129.1 (7.4)	122.5 (5.0)	115.4 (4.7)	121.6 (5.5)
Serum creatinine, micromole/L	278.7 (42.8)	255.8 (39.6)	175.6 (16.8)	258.5 (30.5)	242.2 (51.2)
eGFR, mL/min per 1.73 m^2^	25.7 (4.8)	26.7 (3.9)	32.9 (3.8)	25.9 (3.6)	27.8 (4.0)
uACR, mg/mmol	45.8 (24.5)	11.2 (5.76)	234.0 (154)	145.6 (77.1)	110.1 (65.3)
uPCR, mg/mmol	236.3 (104.5)	117.5 (68.1)	20 (0.5)	164 (94.6)	104.3 (67)
C-reactive protein, mg/L	8.8 (3.3)	5.6 (1.9)	7.7 (2.9)	4.6 (1.0)	6.7 (2.3)
Albumin, g/L	34.6 (3.7)	37.1 (3.2)	35.2 (3.8)	32.6 (3.5)	34.88 (3.55)

Mean values and the standard error of the mean for each of the four intravenous iron groups (*n* = 10 per group)

*Abbreviations*: *eGFR* estimated glomerular filtration rate, *uACR/uPCR* urinary albumin:creatinine ratio/urinary protein:creatinine ratio

**Table 4:** Etiology of renal disease in whole cohort of patients

	Cosmofer 200	Venofer 200	Monofer 200	Monofer 1000	Total
Diabetes	2	4	4	3	13
Hypertension	2	1	2	5	10
Polycystic kidney disease	0	2	2	1	5
Pyelonephritis	0	1	0	1	2
Glomerulonephritis	2	0	2	0	4
Other	4	2	0	0	6

## Discussion

The relative safety of IV iron preparations regarding acute infusion-related effects is not well characterized. A few retrospective epidemiological studies have explored the relative risks of serious adverse events associated with IV iron products. A main knowledge gap in the literature is the lack of head-to-head randomized controlled studies comparing IV preparations; thus, true comparative analysis is difficult. Therefore, further studies are needed. This small randomized open-label study will provide valuable information for clinicians to consider.

## Conclusions

The IRON-CKD study will provide further important clinical and experimental data to allow some evaluation of the relative acute effects associated with Venofer, Cosmofer, and Monofer (low- and high-dose) on markers of oxidative stress, inflammation and acute kidney injury and potentially differences in the iron preparations acknowledging the small numbers. The data will be hypothesis-generating. In addition, data will be generated on the relative efficacy of these agents for improving Hb concentrations and again this may suggest simply a possible difference. Finally, the study will examine measures of endothelial function and quality of life among patients with non–dialysis-dependent CKD. Recent publications have shown variations in effects and this study will add further clinical and mechanistic data to allow one to differentiate the iron products. The future use of the data from this study may allow one to power/design a larger study which will permit investigators to compare these iron preparations in a more statistically and clinically meaningful fashion.

## Abbreviations

AKI: Acute kidney injury
CKD: Chronic kidney disease
CRP: C-reactive protein
EDTA: Ethylenediaminetetraacetic acid
eGFR: Estimated glomerular filtration rate
Hb: Hemoglobin
IV: Intravenous
LPI: Labile plasma iron
NGAL: Neutrophil gelatinase–associated lipocalin
NHS: National Health Service
PWV: Pulse wave velocity
RES: Reticulo-endothelial system
SEM: Standard error of the mean
SF: Serum ferritin
SF-36: 36-Item Short Form Health Survey
TS: Transferrin saturation
uACR: Urinary albumin:creatinine ratio
uPCR: Urinary protein:creatinine ratio
