# Correction to: Protocol and baseline data for a prospective open-label explorative randomized singlecenter comparative study to determine the effects of various intravenous iron preparations on markers of oxidative stress and kidney injury in chronic kidney disease (IRON-CKD)

**DOI:** 10.1186/s13063-020-04238-w

**Published:** 2020-03-17

**Authors:** Ahmed Ziedan, Xenophon Kassianides, Sunil Bhandari

**Affiliations:** Hull University Teaching Hospitals NHS Trust and Hull York Medical School, Hull Royal Infirmary, Anlaby Road, Hull, HU3 2JZ UK

**Correction to: Trials**

**https://doi.org/10.1186/s13063-019-3291-x**

After publication of our article [1], the database of IRON-CKD has undergone vigorous inspection – the authors have therefore re-examined database. On final cleaning of the database after completion of the study for all the participants it was noticed that transcription errors in data entry from the case report files (CRFs) and also from laboratory results occurred. Therefore, to ensure complete independence and as quality check of the whole data set a separate team was formed, composed by members of the research team and the research and development department, the sponsor. The database was restructured and re-audited four times; data and dates of blood investigations were noted and correlated with evidence in the medical notes (the source documents). Only evidence arising from laboratory and equipment with tracking memory (described as “hard” evidence) was used in the final dataset for the baseline data and the final future analysis. Additionally, initial “missing” values have also been entered from the source data, where available, to ensure the completeness of the dataset. Therefore, the mean values and SEM have been recalculated based on the revised complete and correct dataset available (Additional File 1) and removal of errors in full agreement with final database which has independently been checked and locked. With this correction, the Abstract section is also modified to the below:

Results: Between October 2015 and April 2018, 521 individuals were identified as potential participants; 216 were contacted, 56 expressed an interest, 49 attended a screening visit, and 40 were confirmed to meet the eligibility criteria and were randomly assigned. The mean age was 58.8 (standard error of the mean 2.2) years, and 23 (58%) were male. All patients were white and English-speaking. The mean SF was 68.8 μg/L, TS was 21.4%, and haemoglobin was 122.6 g/L at randomization for the whole group. The mean estimated glomerular filtration rate was 28.2 mL/min/1.73 m2 the urinary protein/ creatinine ratio was 154.2 mg/mmol, and CRP was 7.5 mg/L.

Revised Table 3:

**Table 3:** Baseline demographic data

	Venofer 200	Cosmofer 200	Monofer 200	Monofer 1000	Total group
Age, years (SEM)	52.1 (4.3)	64.2 (2.6)	59.5 (5.0)	59.3 (5.3)	58.8 (2.2)
Male/female, numbers	6/4	6/4	4/6	7/3	23/17
Serum ferritin, μg/L (SEM)	58.5 (11.6)	82.9 (21.8)	64.7 (10.9)	69.1 (18.4)	68.8 (8.0)
Transferrin saturation percentage (SEM)	21.1 (2.9)	25.4 (4.9)	21.2 (3.1)	17.8 (1.7)	21.4 (1.7)
Haemoglobin, g/L (SEM)	122.1 (4.6)	129.6 (7.5)	123.0 (5.5)	115.5 (4.5)	122.6 (2.8)
Serum creatinine, micromole/L (SEM)	279.2 (42.7)	247.6 (39.3)	176.2 (16.8)	235.5 (22.5)	234.6 (16.6)
eGFR, mL/min per 1.73 m^2^ (SEM)	25.8 (5.1)	26.3 (4.3)	34.8 (4.2)	26.2 (3.9)	28.2 (2.2)
uACR, mg/mmol (SEM)	74.1 (33.1)	26.4 (8.8)	167.0 (110.8)	144.6 (50.5)	107.9 (33.9)
uPCR, mg/mmol (SEM)	105.9 (47.3)	37.7 (12.6)	238.6 (158.3)	206.5 (72.2)	154.2 (48.4)
Serum Albumin g/L (SEM)	36.5 (1.02)	38.0 (1.48)	36.3 (1.56)	35.1 (1.27)	36.5 (0.67)
C-reactive protein, mg/L (SEM)	8.1 (3.3)	5.2 (1.8)	9.7 (4.8)	6.8 (2.6)	7.5 (1.6)

Baseline characteristics of randomly assigned participants:

In total, 40 patients have been randomly assigned. The mean age was 58.8 years (standard error of the mean (SEM 2.2.) and 23 (58%) were male (Table 3). The mean SF was 68.8 μg/L (SEM 8.0), TS 21.4% (SEM 1.7), and Hb 122.6 g/L (SEM 2.8) at randomization for the whole group. The mean eGFR was mL/min/1.73m^2^ (SEM 2.2), uACR 107.9 mg/mmol (SEM 33.9), and uPCR 154.2 mg/mmol (SEM 48.4). CRP was 7.5 mg/L (SEM 1.6).

The authors have also notified us of missing Xenophon Kassianides from the original authorship list. The need to change the authorship resulted from the work of Dr. Xenophon Kassianides in verifying and learing the data, identifying the previous omissions and re-designing the tables needed to be added in the publication. Dr. Kassianides was responsible for the creation and review of final dataset alongside the statistical analysis (means analysis) of baseline values.

The Author Contribution and Competing Interestes sections therefore change to the below:

- Author contributions

AZ contributed to this manuscript and approval of this submission and wrote the first draft of the study protocol. XK was involved in final dataset formation, review and analysis of results. SB participated in all aspects of the study, obtained funding for the study, and critically revised the manuscript.

The order of authorship has been a joint decision of the co-authors based on substantial contribution to conception and design, execution, analysis, and interpretation of data. SB is the senior author. All authors read and approved the final manuscript.

- Competing interests

AZ declares that he has no competing interests. XK declares that he has no competing interest. SB has received honorarium for lectures, attended expert opinion committees, and received educational funds to attend the European Society of Dialysis and Transplantation (EDTA annual scienctic meeting 2016 and 2017) from Pharmacosmos and Vifor Pharma.
